# An Experimental Test of Condition-Dependent Male and Female Mate Choice in Zebra Finches

**DOI:** 10.1371/journal.pone.0023974

**Published:** 2011-08-25

**Authors:** Marie-Jeanne Holveck, Nicole Geberzahn, Katharina Riebel

**Affiliations:** Behavioural Biology Group Institute of Biology (IBL), Leiden University, Leiden, The Netherlands; Auburn University, United States of America

## Abstract

In mating systems with social monogamy and obligatory bi-parental care, such as found in many songbird species, male and female fitness depends on the combined parental investment. Hence, both sexes should gain from choosing mates in high rather than low condition. However, theory also predicts that an individual's phenotypic quality can constrain choice, if low condition individuals cannot afford prolonged search efforts and/or face higher risk of rejection. In systems with mutual mate choice, the interaction between male and female condition should thus be a better predictor of choice than either factor in isolation. To address this prediction experimentally, we manipulated male and female condition and subsequently tested male and female mating preferences in zebra finches *Taeniopygia guttata*, a songbird species with mutual mate choice and obligatory bi-parental care. We experimentally altered phenotypic quality by manipulating the brood size in which the birds were reared. Patterns of association for high- or low-condition individuals of the opposite sex differed for male and female focal birds when tested in an 8-way choice arena. Females showed repeatable condition-assortative preferences for males matching their own rearing background. Male preferences were also repeatable, but not predicted by their own or females' rearing background. In combination with a brief review of the literature on condition-dependent mate choice in the zebra finch we discuss whether the observed sex differences and between-studies differences arise because males and females differ in context sensitivity (e.g. male-male competition suppressing male mating preferences), sampling strategies or susceptibility to rearing conditions (e.g. sex-specific effect on physiology). While a picture emerges that juvenile and current state indeed affect preferences, the development and context-dependency of mutual state-dependent mate choice warrants further study.

## Introduction

Given enough variance in quality of mates, mate choice should increase fitness over random mating both in males and females [1], [2], [3]. Theory posits that optimal mate choice cannot be seen independently of the state or condition of the choosing individual. The net balance of benefits and costs of choice such as time and energy loss from search and competition is expected to differ for individuals in high and low condition [4]. Females could employ different state-dependent strategies such as changing the way they rank males (i.e. their preference function), alter their sampling rules (i.e. how they gather information about prospective mates) or adjust the time and effort they invest into realizing a particular preference (‘choosiness’ sensu [5]). Depending on species' ecology and life history these different components are expected to be affected in different ways [5], [6], [7], [8].

More specifically, optimality models of state-dependent mate choice predict reduced sampling effort or choosiness in low-quality individuals if they a) cannot physically afford the costs of prolonged mate search, and/or b) are less successful in competing with their own sex, and/or c) are less successful in attracting the opposite sex, or d) are more likely to be deserted by their mate [1], [9], [10], [11], [12], [13]. However, if the costs of targeting the best mates are sufficiently high (e.g. when such mates are particularly rare), then low-quality individuals might minimize the costs of choice and lost breeding opportunities by changing the direction of their preferences towards low-quality individuals [9], [11].

We recently reported such a quality-assortative preference in female zebra finches *Taeniopygia guttata* [14] that were allowed to choose between the songs of males in different condition. Adult male and female phenotypic quality had been experimentally manipulated by rearing nestlings in experimental broods with few or many siblings. Such brood-size manipulations are known to affect adult physiology, morphology and behavior both in wild and domesticated zebra finches, such that birds from smaller broods fare better than those from large broods (e.g. [15], [16], [17]). Instead of showing a uniform preference for the males of superior quality from small broods, only females that originated from small broods (i.e. that were of high phenotypic quality themselves) preferred the songs of males from small broods. Females reared in large broods, i.e. of lower phenotypic quality, preferred the songs of low quality males that originated like themselves from large broods. Moreover and likewise in agreement with predictions from models of state-dependent mate choice [9], [11], in a later phase of the experiment, quality-matched pairs also showed a much shorter latency to breeding than non-matched pairs, suggesting a reproductive advantage arising from quicker pair formation [14], [18].

**Figure 1:**
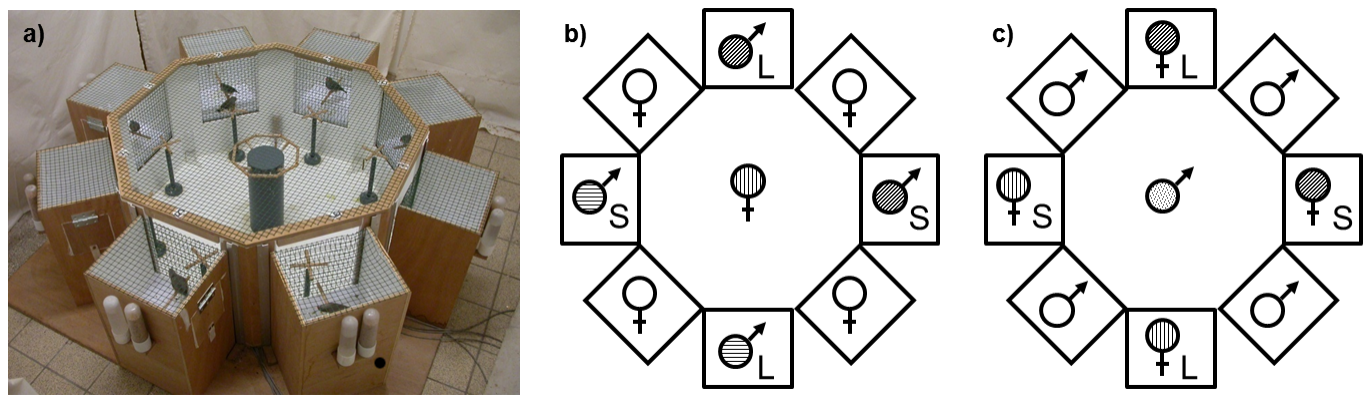
Testing apparatus. a) Photograph of apparatus. b) Plan view illustrating a test with a focal female or c) focal male in the central compartment. Stimulus birds were presented with four same-sex birds randomly picked from the breeding stock (open symbols) and 4 opposite-sex birds from manipulated brood sizes (♂_L_, ♀_L_ = male, female from a large brood; ♂_S_, ♀_S_ = male, female from small brood; filling patterns symbolize tutoring-group ID). Half-way through the 8h-testing period, stimulus birds (staying within their holding cages) were rotated 180 degrees. For new trials with a new stimulus set, the start position of stimulus categories were rotated one position clockwise (see methods).

The tests in [14] took advantage of the fact that song preferences are an important predictor of female mate choice in zebra finches (for review see [19]). Testing female song preferences allows testing their mating preferences without male choice behavior as confound of choice. However, while establishing preference is an important step in understanding female mating decisions, it is also important to keep in mind that preference is but one component of mate choice [5], [20]. To realize a particular preference females also have to search for, find and be accepted by their preferred male [5], [6]. In the operant song preference test in [14] female choice was unconstrained: females could choose how much song they wanted to hear independently of male courtship motivation and choice simply by key pecking. However, male zebra finches have been reported to prefer females in better condition [21], [22], [23] (but see [15], [24]), raising the question of whether the condition-assortative preference we observed in an unconstrained choice situation would hold if several live males and females of different quality were simultaneously present. Such a socially more complex situation could modify the previously observed assortative choices because of the larger option set [25], [26], feedback between male and female courtship behavior [19] and effects of same-sex competitors on decision making [27]. We therefore decided to test the males and females from the brood-size manipulation experiment in a multiple, interactive choice situation. Focal male and female birds could express their preferences in an 8-way choice arena by perching near same- or opposite-sex individuals housed in individual compartments grouped around a central arena (see Figure 1). The apparatus had been designed and successfully used for male preference tests as part of an imprinting study in our lab [28] and similar 4- to 10-way choice arenas have previously been used to test male and female preferences in zebra finches (e.g. [29], [30], [31], [32]). This set-up thus enabled us to test both sexes for effects of manipulated juvenile condition on adult mating preferences in the same context and with the same method.

Using this 8-way choice arena, we first tested the condition-dependency and repeatability of female mating preferences: the females from the earlier operant song preference tests [14] were now allowed to choose between the four live males from large and small brood sizes whose songs they had been tested with 6 months earlier (see Table 1). Instead of the unconstrained choice situation of the operant task where females could unlimitedly choose between two songs, in the choice arena females were now presented with a larger option set as well as same-sex competitors. Moreover, males and females could now vocally and visually interact and females could therefore receive visual and behavioral information on the males not available in a song preference test. In a second experiment, we likewise tested condition-dependency of male mating preference. Unlike the females, males had not been tested for song preferences prior to this experiment (because female zebra finches do not sing), but the setup allowed us to test mating preferences of males that like the females originated from experimentally manipulated small or large broods.

**Table 1:** Schematic view of the time course of experimental manipulations and preference tests.

Age^a^ (mean ± 1 SD)	Experimental phase	Description
0 to 3±2	Hatchlings with biological parents	113 hatchlings in 30 pairs
3±2 to 34±3	Cross-fostering of chicks	59 chicks in 19 small broods (2-3 chicks), 54 chicks in 11 large broods (5-6 chicks)
34±3 to 69±3	Song tutoring (4 chicks + adult pair)	17 groups×4 chicks
From 69±3	Housing in same-sex peer groups	Single-sex mixed-treatment groups (4-5 birds)
164±15	Female song preference test	24 ♀♀ and songs of 26 ♂♂ [14]
270^b^	Morphological measures	All experimental birds
402±11	Standard metabolic rate	43 birds born in 2004 [38]
♀♀: 330±35, ♂♂: 428±31	Mate preference test	42 focal birds: 24 ♀♀, 18 ♂♂; 119 stimulus birds: 44 experimental (20 ♀♀ and 24 ♂♂), 75 from breeding stock (43 ♀♀ and 32 ♂♂)
487±63	Experimental breeding	24 ♀♀ and 24 ♂♂ [14]

a Days post-hatching.

b Mean age per brood.

We hypothesized that if females' song preferences [14] indeed predict their preferences for live males [33], and if these are in line with the predictions of state-dependent mate choice theory [9], [11], then females should also show quality-assortative preferences for live males. Predictions on the effect of male condition on the direction of male preferences cannot be based on earlier empirical findings as this is to our knowledge the first experimental study on how experimentally altered developmental condition might affect avian male mating preferences. However, based on theoretical considerations we should expect that in species with obligatory biparental care both males and females ought to be choosy [34], [35]. In line with this, male zebra finches (of unmanipulated condition) have been reported to prefer (manipulated) high-condition females [21], [22]. Our experimental manipulation of male condition allows us to test whether male condition can explain additional variation in male mating preferences as predicted by models of state-dependent mutual mate choice [1], [9], [10], [11], [12], [13].

## Materials and Methods

### Ethics Statement

This study was conducted with domesticated zebra finches from the breeding colony at the Institute of Biology at Leiden University (for details on the genetic background of this population, see [36]). All procedures followed Dutch laws and were approved by Leiden University's Animal Experimentation Committee (Dierexperimentencommissie Universiteit Leiden, permit DEC 04090).

### Birds and housing

All birds tested for mating preferences originated from a brood-size manipulation experiment [14], [37] that had significantly affected adult phenotypes: birds from small broods were of significantly larger structural size and had a higher metabolic efficiency than birds from large broods [38]. Males from small broods had song that was more similar to their tutors' song and more consistent in performance than song from males from large broods [37] and females' song preferences were assortative with respect to their own rearing brood size [14]. Birds were housed in indoor bird rooms at Leiden University in standard laboratory cages (80×40×40 cm) on a 13.30∶10.30 light∶dark schedule (lights on 07:00–20:30 Central European Time) at 20–22°C and 35–50% humidity. Cages had solid side walls and were stacked three cages high in three rows along the length of the room and with other birds 2 m across the aisle. Throughout, birds had *ad libitum* access to a commercial tropical seed mixture (Tijssen, Hazerswoude, Holland), drinking water and cuttlebone. This was supplemented thrice weekly with egg food (Witte Molen, B.V., Meeuwen, Holland), twice with millet branches and once with germinated seeds.

Our original brood-size manipulation experiment [14], [37] had cross-fostered the chicks of first time breeders in small (2–3 chicks per nest) or large broods (5–6 chicks) in two breeding rounds in two different years (2004 and 2005). Chicks were housed with their foster parents until nutritional independence at 33.5±3.1 (mean±1 SD) days post-hatching. At this age, 68 of the young birds were regrouped into 17 different ‘song-tutoring groups’. Each group consisted of four chicks from different hatching nests and foster broods plus one unrelated adult male (the tutor) and his female mate. This way, we obtained same-sex matched pairs of different rearing background (one from a large and one from a small brood, referred to as ‘L-S sets’ in the remainder of the text) but which had learned their songs from the same tutors. Hence, birds in L-S sets were always ‘different rearing background, but same song culture’. This way we could control for learned song preferences when testing effects of condition on male attractiveness and female mating preferences [14]. In the tutoring groups, there were two types of sex*treatment combinations. Mixed-sex tutoring groups (*n* = 13 groups, 52 fledglings) consisted of two male and two female fledglings: one from each sex and brood size. Male-only tutoring groups (*n* = 4 groups, 16 fledglings) consisted of four male fledglings each: two from a small and two from a large brood. With the end of the sensitive phase for song learning at 69.4±3.0 days post-hatching [19], [39], [40], tutoring groups were split up and from then on housed in single-sex groups of 4–5 birds. Birds in such ‘peer-groups’ always originated from different brood sizes and different tutor groups. Mate choice experiments started when females were 330±35 (*n* = 24) and males 428±31 (*n* = 18) days old. As summarized in Table 1, prior to the experiments described here all females (at 164±15 days) had already undergone the song preference tests described above [14] and the birds from the 2004 breeding episode (29 males and 14 females) had also been measured for standard metabolic rate when 402±11 days old [38]. At 270 days post-hatching, we measured tarsus length (±0.05 mm) with calipers as an index of structural size and body mass (±0.1 g Sartorius BL600 scale). Experimental birds from large broods had shorter tarsi than those from small broods (males from large broods: mean±1 SD = 15.0±0.6 mm, small broods: 15.4±0.5, χ^2^_1_ = 5.08, *P* = 0.024; females from large broods: 15.2±0.5 mm, small broods: 15.6±0.4, χ^2^_1_ = 3.65, *P* = 0.056; *n* = 21, 21, 12 and 13 respectively; linear mixed models with year, hatching nest, foster brood, and tutoring group as random factors) but did not differ in absolute mass (males from large broods: 18.6±2.4 g, small broods: 17.9±2.7 g; females from large broods: 17.7±2.3 g, small broods: 17.4±2.2 g) or size-corrected mass (calculated as standard residuals of the linear regression of mass on tarsus size for males and females separately; all 0.00≤*Χ* ^2^_1_≤0.71, 0.4≤*P*≤1.0). Stimulus males (in experiment 1) and focal males (in experiment 2) (*n* = 24 and 18 respectively, see description below) did not differ significantly in tarsus length, absolute and size-corrected mass at 270 days post-hatching (all 0.00≤*Χ* ^2^_1_≤0.83, 0.4≤*P*≤1.0).

### Apparatus and acclimatization period

All birds were tested in the same 8-way choice apparatus (see Figure 1a) surrounded by floor-to-ceiling beige cotton screens. It consisted of a wire-mesh octagonal cage (⌀ 84 cm) with eight small removable cages attached to its sides (height×width×depth outside 35×26×26 cm, inside compartment 25×25×25 cm) with only the top and upper front half made from wire-mesh. A focal bird could thus not see the stimulus birds from the floor, but watch and hear all stimulus birds from the ring shaped perch (⌀ 20 cm) on the central pillar or watch individual birds from a cross-shaped perch (10 cm) in front of each stimulus birds' cage (and still hear all other birds). Stimulus birds could see the focal bird when it was perched on the central pillar or on the perch in front of them. On their perches, stimulus birds could hear the other birds but not see the stimulus bird directly opposite as the central pillar blocked the view across. Likewise, adjacent neighbors were at invisible angles (except when clinging to the wire mesh of their cages), but they could see and were visible to the two stimulus birds on either side of the one directly opposite (see Figure 1).

Focal birds were placed in the central cage between 17:00 and 18:30 hours (at least 2 hours before lights off) the day before a test to acclimatize. At this stage, opaque screens blocked the view to the (absent) side cages. Stimulus birds were simultaneously acclimatized to the small attachable side cages but in an adjacent room. They were moved within these cages the next morning 1 hour before their test started at 9:00 (2 ours after lights went on) to the testing room and all eight were placed adjacent to each of the 8 side walls. Testing and data registration started when the opaque dividers were removed at 9:00 h and the stimulus birds were visible to the focal bird. Each test terminated at 17:00 h and thus lasted a total of 8 hours, but half-way through, the experimenter replaced the plastic partitions between the central cage and the eight side cages and all stimulus birds were switched with the bird in the position directly opposite their own. During this manipulation the data registration was stopped, but switched on again when the plastic partitions were removed and the test resumed. The time required to move the stimulus birds (mean±1 SD = 00:04±00:02 h, *n* = 42) was subtracted from the total experimental time (08:02±00:02 h). This actual total testing time (07:58±00:02 h) was used for further analyses. Between tests, all cages were cleaned and food and water containers refilled each time before a new bird was introduced.

### Mate-choice trials

After acclimatization, the opaque screens were lifted and the focal bird (male or female) in the centre of the arena could now see the eight stimulus birds in the small side cages (Figure 1) and choose to approach them. The four opposite-sex stimulus birds always were two matched L-S sets from a different natal- and foster-nest and tutoring group as the focal bird. All same-sex stimulus birds were non-experimental birds from our breeding stock and unrelated and unfamiliar to the focal bird (*n* = 43 females and 32 males, all at least 5 months old). Stimulus birds were placed around the central arena alternating males and females and brood-size treatment (Figure 1b–c), rotating one position clockwise with each new set of 8 stimulus birds. Each L-S set of focal birds was tested on subsequent days with the same unique set of 8 stimulus birds in identical positions, fully balancing whether the small- or large-brood bird got tested first. A total of 24 females and 26 males from mixed-sex tutoring groups (i.e. the complete sample of 12 female and 13 male matched L-S sets from [14]) and an additional 18 males (in 9 L-S matched pairs, 16 males from male-only tutoring groups and 2 males from a mixed-sex tutoring group) that up until now had not been involved in prior tests were still available from the original brood-size experiment. For each choice test we made sure that 1) every focal bird could choose between four opposite-sex experimental subjects of two song-culture matched L-S sets; 2) that every focal individual had not been a stimulus bird before and was hence naïve with respect to the testing arena and other individuals in the arena; 3) that there were never birds from the same natal or foster nest within the same set of stimulus birds. In experiment 1, the 24 females from our earlier operant song preference test [14] got a choice between stimulus males whose song they had been previously tested with (*n* = 24 males).

In experiment 2, the additional 18 experimental males that had not previously been used as stimulus birds in the arena were tested as focal birds. Because we had more experimental males than females the L-S females from experiment 1 were now used as stimulus birds. This was on average 80±11 (mean±1 SD) days after they had been focal birds in experiment 1.

### Data registration

The perches in front the stimulus birds were equipped with micro-switches connected to a custom-built minicomputer that logged the time of focal subjects' arrivals and departures at each perch. This allowed calculating the number and duration of visits to each stimulus bird. Time spent in front of an opposite-sex stimulus birds in a similar choice arena (10-way choice) has been shown to predict pair formation in aviaries [31]. A test was considered successful if the focal bird had spent at least 60 min with the eight stimuli, and all eight stimuli were visited at least once (39 tests out of 42 tests fulfilled these criteria). Two birds were retested once and one twice after not reaching these criteria. A fourth bird had to be retested since downloading the data failed. Repeats took place between 2 and 9 days after the failed test.

### Statistical analyses

We used both the total amount of time and the number of visits to a particular stimulus bird as response variables because they were not significantly correlated (females: Pearson *r*_22_ = −0.07, *P* = 0.7; males: *r*_16_ = 0.28, *P* = 0.3). In the analyses that tested whether preferences were assortative by brood size, we worked with the proportion of perching time (or number of visits) with opposite-sex stimulus birds from small (versus large) brood sizes as response variable.

We analyzed data with two-tailed (*α* = 0.05) linear mixed models following arcsine or Box-Cox transformations to achieve normality when needed (Shapiro-Wilk tests on linear model residuals: all *P* > 0.05) in R v. 2.12.0 under the lme4 package [41]. We always started with the full model and used a stepwise backward selection procedure on fixed factors until reaching the minimal adequate model. We always kept the model with the best fit based on the log-likelihood ratio test. We report the estimate (± 1 SE) of each fixed factor together with the Chi-statistics of the comparison between the models with and without the tested factor. In analyses, we fitted the focal birds' brood size as fixed factor and year, hatching nest, foster brood, and tutoring group for focal females or matched pair nested within tutoring group for focal males as random factors (see detailed R script in footnotes of Tables 2 and 3). The focal birds' sex was fitted in interaction with brood size in those analyses where male and female data were pooled. We included test order (i.e. whether the first tested bird of a dyad was from a small or large brood; see details above) as a fixed factor in the initial full models, but it did not have significant effects (statistics not shown) and was therefore omitted in subsequent analyses. We checked whether subjects had consistent mating preferences by testing whether their preference strength for opposite-sex stimulus birds from small (versus large) brood sizes in the first testing half correlated with their one in the second testing half. We calculated within-subject repeatability of preferences as the estimates *R*±1 SE following [42] and [43]. One female and male from small-broods each had died before the mate preference tests. They were replaced by other small brood-size individuals, meaning that the design remained balanced with respect to matching small and large brood sizes but in one male L-S set two males had not learned their song from the same tutor. When analyses were run excluding the trials with the replacement birds the results did not change qualitatively (not reported here), hence we report only the analyses of the complete data set.

**Table 2:** Effects of experimental brood size of focal males and females (in separate analyses) on their association patterns with stimulus birds.

Choices for stimulus birds	Response variable	Means±1 SD of focal birds from		Estimate (1 SE)	χ^2^_1_	*P*
		large broods	small broods			
**Focal females^a^ (** [Table-fn nt103] ***n*** ** = 24)**						
All (males and females)	Time proportions	0.86±0.10	0.82±0.12	-0.060 (0.063)^d^	0.96	0.3
	Visit numbers	1250±789	1045±706	-4.604 (5.776)^e^	0.70	0.4
Males vs. females	Time proportions	0.53±0.24	0.58±0.21	0.053 (0.092)	0.36	0.5
	Visit proportions	0.46±0.08	0.51±0.07	0.043 (0.031)	2.01	0.2
Males from small vs. large broods	Time proportions	0.41±0.14	0.61±0.20	0.205 (0.073)	7.29	0.007
	*Time proportions* c			*0.249 (0.158)*	*2.48*	*0.1* c
	Visit proportions	0.48±0.10	0.54±0.10	0.054 (0.047)	1.41	0.2
**Focal males^b^ (** [Table-fn nt103] ***n*** ** = 18)**						
All (males and females)	Time proportions	0.82±0.11	0.82±0.08	-0.006 (0.043)	0.07	0.8
	Visit numbers	1418±574	1331±756	-8.834 (16.086)^f^	0.37	0.5
Females vs. males	Time proportions	0.67±0.18	0.65±0.23	-0.042 (0.072)	0.40	0.5
	Visit proportions	0.59±0.11	0.60±0.16	-0.047 (0.099)^g^	0.26	0.6
Females from small vs. large broods	Time proportions	0.54±0.20	0.45±0.19	-0.073 (0.094)	0.65	0.4
	Visit proportions	0.55±0.12	0.45±0.20	-0.096 (0.081)	1.51	0.2

Models in R script were ^a^lmer(response variable∼brood size+(1|hatching nest)+(1|foster brood)+(1|tutoring group)+(1|year)); ^b^lmer(response variable∼brood size+(1|hatching nest)+(1|foster brood)+(1|tutoring group/matched pair)+(1|year)).

c Model including song preferences in the operant test as a covariate (see text for details).

Transformations were^ d^arcsine or Box-Cox by a factor λ = 0.45^e^, 0.606^f^ and -0.238^g^.

**Table 3:** Comparison of focal males' and females' association patterns with stimulus birds (response variables are as in Table 2).

Stimuli	Response variable	Model terms	Estimate (1 SE)	χ^2^_1_	*P*
All	Time proportions^b^	Brood size	-0.031 (0.046)	0.56	0.5
		Sex	-0.019 (0.047)	0.29	0.6
		Brood size*Sex	0.049 (0.078)	0.43	0.5
	Visit numbers^c^	Brood size	-6.941 (6.796)	1.08	0.3
		Sex	13.850 (10.464)	1.75	0.2
		Brood size*Sex	3.134 (13.595)	0.08	0.8
Opposite vs. same sex	Time proportions^b^	Brood size	-0.060 (0.101)	0.24	0.6
		Sex	0.068 (0.066)	1.13	0.3
		Brood size*Sex	-0.078 (0.124)	0.45	0.5
	Visit proportions^d^	Sex	0.196 (0.060)	9.01	0.003
		Brood size	0.044 (0.069)	0.43	0.5
		Brood size*Sex	-0.108 (0.125)	0.87	0.3
Opposite sex from small vs. large broods	Time proportions	Brood size	0.236 (0.077)	3.08^a^	0.004^a^
		Sex	0.139 (0.075)	1.85^a^	0.1^a^
		Brood size*Sex	-0.323 (0.092)	7.12	0.008
	Visit proportions	Brood size	0.102 (0.064)	1.58^a^	0.1^a^
		Sex	0.078 (0.043)	1.84^a^	0.07^a^
		Brood size*Sex	-0.205 (0.063)	6.52	0.011

a *t*-value and Pr(>|*t*|) are given.

Models in R script were lmer(response variable∼brood size*subject sex+(1|hatching nest)+(1|foster brood)+(1|tutoring group/matched pair)+(1|year)). Transformations were^ b^arcsine or Box-Cox by a factor λ = 0.537^c^ and -0.125^d^.

## Results

### Experiment 1: Testing female mating preferences

**Figure 2:**
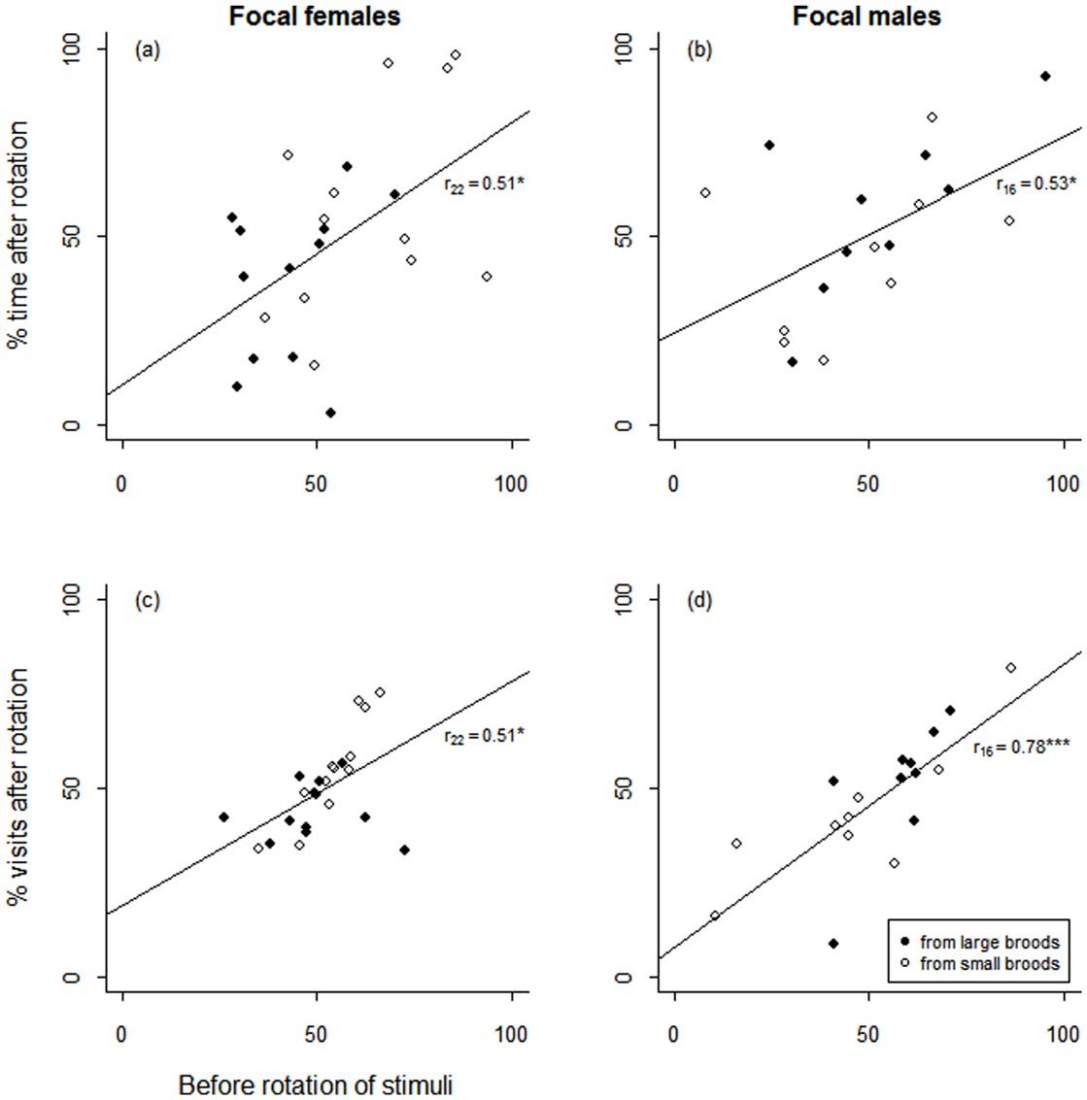
Consistency of preferences after stimulus rotation. Percentages of the total time (a, b) with and total number of visits (c, d) to small-brood opposite-sex stimuli before and after stimulus rotation. Trend lines are y = x and correlation values are Pearson *r.* *P < 0.05, ***P < 0.001.

Focal females spent on average 6:43±00:52 (mean±1 SD) hours: minutes of the total testing time (07:57±00:01 h) perching in front of the stimulus birds (range = 04:16–07:52, *n* = 24) and visited on average 1147±740 times the different stimulus birds during this time (range = 106–3026). There was no significant effect of brood size on the proportion of perching time or on the number of visits to stimulus birds overall, nor on the proportions of perching time or visits to opposite-sex stimulus birds only (all 0.36≤Χ^2^_1_≤2.01, 0.2≤*P*≤0.5, *n* = 24; Table 2). Female preference strength for males from small broods during the second half of the experiment, i.e. after stimulus birds had been rotated to new positions, was consistent with the first half (time proportions: Pearson *r*_22_ = 0.51, *P* = 0.01; visit proportions: *r*_22_ = 0.51, *P* = 0.01; Figure 2a, c).

**Figure 3:**
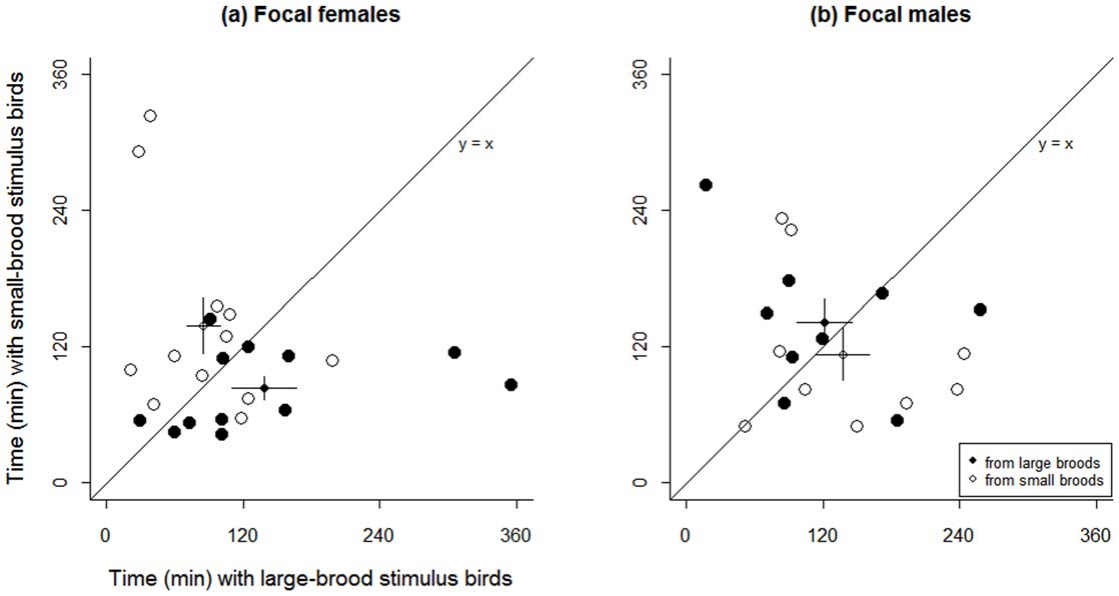
Preference for large and small brood birds by males and females from large and small broods. Total time spent with small-brood versus large-brood opposite-sex stimuli by focal females (a) and males (b) from small and large broods. The y = x line illustrates an equal preference for birds from small and large broods: above this line birds from small brood are preferred, below birds from large broods are preferred. Smaller dots show the means±1 SEM per focal birds' brood sizes. The amount of time spent with opposite-sex birds from small broods depended on the brood size and sex of the focal birds: females from small broods spent significantly more time with males from small broods and females from large broods with males from large broods (see main text for statistical details).

To test whether females showed assortative preferences for quality-matched birds, we compared the proportion of perching time with opposite-sex birds from small brood sizes for females from the two treatments. Females from small broods spent proportionally more time near males from small broods than females from large broods that preferentially associated with males from large broods (*Χ* ^2^_1_ = 7.29, *P* = 0.007, *n* = 24; Figure 3a; Table 2). Female preferences assessed by the proportion of visits to small-brood males out of visits to all males showed a similar, but not significant, trend (*Χ* ^2^_1_ = 1.41, *P* = 0.2, *n* = 24; Table 2).

### Repeatability of female preference across contexts

In the tests presented here, each female was simultaneously tested with the four males whose songs she had been tested with several months before in two different binary operant song preference tests (each presenting songs of two males from one L-S set [14]). This provided us with the unique opportunity to test whether females' quality-assortative preferences were repeatable over time and between substantially different contexts. Females' preferences measured as count-based proportions in the song preference test (sum of key pecks for small-brood males divided by the total pecks in the two sequential operant tests) and measured as the proportion of perching time for the two small-brood males in the mate preference test were indeed repeatable (*R*±1 SE = 0.44±0.17, *F*_23,24_ = 2.5, *P* = 0.01).

A comparison of the preference scores in the song preference test of the earlier study with the preference scores in the live bird test in the choice arena also provided us with the opportunity to assess whether female choices based on male condition would be stronger when they were more experienced and when they could base their choices not only on acoustic cues but also on visual cues. Neither of these factors affected female choice on top and above the acoustic cues: when re-running our analyses controlling for song preferences in the operant test (by adding it as a covariate), the effect of female brood size on the proportion of perching time with males from small brood sizes was not significant anymore (*Χ* ^2^_1_ = 2.48, *P* = 0.1, *n* = 24; Table 2).

### Experiment 2: Testing male mating preferences

Focal males spent on average 6:32±00:44 hours: minutes (range = 05:19–07:46, *n* = 18) of the total testing time (07:58±00:03 h) on the perches in front of the stimulus birds (range = 05:19–07:46, *n* = 18) and visited 1375±652 times the 8 stimulus birds during this time (range = 152–2974). In males, like in the females in experiment 1, the strength of preferences for opposite-sex stimulus birds from small broods during the second half of the experiment after stimulus birds had been rotated to new positions was consistent with the first half (time proportions: Pearson *r*_16_ = 0.53, *P* = 0.02; visit proportions: *r*_16_ = 0.78, *P* < 0.001; Figure 2b, d).

In focal males, there was no significant effect of brood size on any of the preference variables (all 0.07≤*Χ* ^2^_1_≤1.51, 0.2≤*P*≤0.8, *n* = 18; Table 2). There was no effect of the brood-size treatment on the proportion of time focal males spent perching near females of either brood sizes *Χ* ^2^_1_ = 0.65, *P* = 0.4; Figure 3b) nor on the proportion of visits to females from small broods from visits to all females *Χ* ^2^_1_ = 1.51, *P* = 0.2; both *n* = 18; Table 2). This result is unlikely to be the outcome of the slightly smaller sample size for males (*n* = 18 vs. *n* = 24 females) as apparent when comparing the low estimate of the effect size and the size of the error in the male sample with the corresponding values in the female sample (Table 2).

### Comparisons of male and female behavior in experiments 1 and 2

Focal males and females did not differ in the proportion of perching time or in the number of visits to stimulus birds overall, nor in the proportion of perching time near opposite-sex stimulus birds only (all 0.29≤Χ^2^_1_≤1.75, 0.2≤*P*≤0.6, *n* = 42; Table 3). However, focal males made a significantly greater proportion of their visits to opposite-sex stimuli than did focal females (*Χ* ^2^_1_ = 9.01, *P* = 0.003, *n* = 42; Table 3). The difference between males and females from small broods was smaller than the difference between males and females from large broods for the proportions of perching time (*Χ* ^2^_1_ = 7.12, *P* = 0.008; Figure 3) and visits (*Χ* ^2^_1_ = 6.52, *P* = 0.011; both *n* = 42) to opposite-sex birds from small broods (see the negative coefficients and significant brood size*sex interactions in Table 3).

## Discussion

Our experimental tests of state-dependency of male and female mating preferences in the mate choice arena showed a treatment effect (experimental brood size) on the direction of female, but not male mating preferences. Female, but not male focal birds showed assortative preferences with respect to rearing condition. Because the direction of female mating preferences was best explained by the combination of their own and males' rearing background, we conclude that the treatment affected males' condition such that it affected female choice. Effects of brood size manipulations on adult phenotype and condition are replicated in several wild and domesticated populations of zebra finches: birds from small experimental brood sizes fare better on various measures of phenotypic quality than birds from large experimental broods [14], [15], [16], [17], [37], [38], [44], [45], [46]. And as established prior to this study, our experimental subjects from large and small brood sizes differed indeed in size and in metabolic efficiency [38], males' song [14], [37] and female song preferences [14]. The brood size manipulation thus caused differences in state which according to theory should affect the expression of sexually selected traits and preferences [4], [9], [47] either of which could affect the process of mutual mate choice.

Our observations that females' and males' state affected female preferences confirm our earlier findings in non-interactive song preference tests [14]. In contrast, the males tested in the same set-up allocated their visiting time independently of female condition. An absence of preference for high-quality females from small brood sizes cannot arise from a general lack of mate choice in male zebra finches. Several studies have demonstrated that males choose too (e.g. [21], [22], [23], [28], [30], [32], [48], [49]) and moreover prefer females in better condition [21], [22], [23]. The males in our study were not indiscriminate either: they showed specific and consistent preferences (based on time or visits) for the different females in their set. Moreover, several studies have successfully used comparable 4- and 10-way mate-choice setups to demonstrate male preferences and the very same set-up we used here has previously demonstrated an imprinted preference for female beak color in males [28]. It is worth pointing out, however, that all previous studies showing male preferences tested single males which were given the choice between either two [21], [22], [23] or several females (e.g. [28], [30], [31], [32]). Our study is the first to test zebra finch males in the presence of other males which makes it possible that the perceived male competition (listening to calls is sufficient in this respect, [50]) had a context specific modulating effect on male preferences. Such effects have been demonstrated in other species, where social feedback (e.g. [51], [52]) and male-male competition can affect male displays and mating preferences in various ways (reviewed in [27]) or even suppress male mating preferences altogether [53].

There has been little research effort into male mate choice and its condition-dependency to date [7], [54], [55] and we cannot conclude at this stage whether the cause of the observed sex differences in behavior resulted from sex differences in context sensitivity (e.g. male-male competition affecting male preferences), sampling strategy or susceptibility to the treatment (e.g. via sex-specific effects of rearing condition on physiological parameters, [38], [56], [57]). The overview provided in Table 4, which lists studies that have manipulated condition in choosing and/or chosen zebra finches, shows that the picture regarding effects of condition on male and female zebra finch mating preferences is far from clear and suggests different effects of different manipulations at different life phases. The variety of treatments and testing contexts across studies makes it difficult to disentangle these factors at the moment.

**Table 4:** Studies with experimental manipulations of male or female condition prior to mate preference tests in zebra finches.

Type of manipulation on birds		Chooser identity		Preference for	Preference test		Effect on choosers				References
Choosers	Stimuli	Age^a^	Sex		Type	# stimuli	D	P	S	A	
Brood size	Brood size	3-35	F	Assortative	CC	4 ♂♂+4 ♀♀	√	no	no	no	This study
			M	Individual		4 ♀♀+4 ♂♂	no	no	no	no	
Brood size	Brood size	3-35	F	Assortative	SB	2 ♂♂ songs	√	no	no	no	[14]
Brood size	--	3-35	F	Individual	SB	2 ♂♂ songs	no	√	no	no	[63]
--	Brood size	(1-)3-50	F	HC (small broods)	CC	2 ♂♂					[15]
			M	No		2 ♀♀					
Feather clipping	Color rings	Adult	F	HC (red rings)	CC	2 ♂♂	no	√	no	no	[24]
			M	No		4 ♀♀					
Food quality^b^	Food quality^b^	Adult	F	HC^b^	CC	2 ♂♂	no	√^c^			[23]
			M	HC^b^		2 ♀♀					
Food quantity	--	5-30	F	Individual	CC^d^	4 ♂♂	no	no	no	√^e^	[64]
--	Brood size	3-35	F	No	CC	2 ♂♂					[65]
--	Color rings	Adult	F	HC (red rings)	NC	1 ♂					[52]
--	Food quality	35-60	F	HC	CC	2 ♂♂					[66]
--	Food quality	Adult	F	HC (carotenoids+)	CC	2 ♂♂					[67]
--	Food quality	Adult	M	HC	CC	2 ♀♀					[21]
--	Food quality	Adult	M	HC	CC	2 ♀♀					[22]

D, direction of preference; P, preference strength; A, activity; S, sampling; M, males; F, females; CC, choice chamber (summarizes any setup where several live stimulus birds could be inspected and approached by a focal bird); SB, Skinner-box; NC, no-choice (one female and one male placed together); HC, high condition; LC, low condition.

a Days post-hatching at manipulation.

b Experimental groups based on pre-treatment mass: top 10 birds = ‘high-condition’ group, remainders = ‘low-condition’ group.

c Low-condition individuals showed more pronounced preferences than high-condition individuals.

d Live males, but no songs.

e Total number of perch hops but not number or type of sampled males affected.

Although we had kept the experimental procedures nearly identical for males and females, focal females had been tutored in mixed-sex tutor groups while most focal males from experiment 2 had been tutored in groups consisting of four young males. Sibling group size and sex ratios in tutoring groups can affect song learning [58], [59] and social and courtship behavior. Although tutoring-group size was controlled and all young birds could observe courtship and imprint on an adult male and his female mate, the early experiences with more male competition could have had an effect on males' behavior in the mate choice arena. On the other hand, our paired design did control for within tutoring-group and between treatment effects on learning: there were always two males of different rearing background that learned the same song under the same circumstances (a matched L-S set) and these two were later tested for preferences with the same 8 stimulus birds. Because tutor group and stimulus set composition were identical for males from small and large brood sizes, either seems unlikely to have caused the absence of a difference in behavior between large and small brood males in the choice arena.

Could females' familiarity with the songs they had heard 2 months previously have affected their preferences for the singers? We cannot exclude this, but assume a minor effect if any, because earlier tests where adult females were exposed to a comparable amount of playback did not make these songs more attractive than unfamiliar songs [60]. Thus, the differences in exposure ratios (smaller in order of magnitude) between preferred and non-preferred songs during the preference tests are even less likely to have made the two preferred (quality-matched) songs more familiar. Moreover, experiments with varying the order of song versus life male tests showed high across context consistency without order effects [33], so we think it most likely that females were attracted to particular singers because of the quality of their song.

A more pronounced condition-dependency of female than male mate choice agrees with theory [1], [3]: the sex showing the higher investment per offspring is expected to be choosier and in birds the substantial cost of egg production is borne by females only. However, most songbirds show social monogamy and obligatory joined brood care – both incubation and feeding of the young are shared by males and females. Under such a scenario, males can improve their fitness by choosing a high-quality mate [1], especially in species with monogamous pair bonds and low rates of extra-pair paternity such as zebra finches [61]. Based on these theoretical considerations, earlier observations of male mate choice (e.g. [21], [22], [23], [28], [30], [32], [48], [49]) and the observed interaction between male and female condition on female preference, we had expected an effect of condition also on male mating preferences but could not observe it in this experiment. Interestingly, (non-experimental) males in another brood size manipulation study [15], did not show a directional preference for females from smaller broods either, whereas (non-experimental) females discriminated between males from small and large broods but with an overall directional preference for small-brood males. The overview provided by Table 4 shows that the only two studies [21], [22] that showed an effect of female condition on male mating preferences in zebra finches did not test effects of early phenotype induction. Instead, female condition was improved just prior to preference tests by a high-quality, high-protein diet that enhanced egg production and fertility. In contrast, the brood-size manipulations in our experiment had no effect on female fertility (measured by egg mass and clutch size, [14]) two months after we tested male preferences. Manipulations of female condition by other authors and by the means of inbreeding also failed to affect egg mass or clutch size [47].

The quality-assortative preferences females demonstrated in the one-way unconstrained song preference tests [14] proved to be repeatable in the different, socially more complex and interactive mating context of an 8-way choice arena. The across-context observation of this effect suggests that quality-assortative or ‘prudent choice’ [11] is a mating tactic not only predicted by theory (for discussion see [62]) but now also demonstrated experimentally. The asymmetry in mating tactics confirms that female preferences might drive much of males' courting effort and eventual choice (for review see [19]). The strong interaction between male and female condition on females' mating decisions further confirms the importance of the early rearing environment on mating decisions and are in line with predictions from state-dependent mate choice theory [1], [9], [10], [11], [12], [13].
